# Development of a prediction model for head and neck volume reduction by clinical factors, dose–volume histogram parameters and radiomics in head and neck cancer^†^

**DOI:** 10.1093/jrr/rrad052

**Published:** 2023-07-18

**Authors:** Miyu Ishizawa, Shohei Tanaka, Hisamichi Takagi, Noriyuki Kadoya, Kiyokazu Sato, Rei Umezawa, Keiichi Jingu, Ken Takeda

**Affiliations:** Department of Radiological Technology, Faculty of Medicine, School of Health Sciences, Tohoku University, 21 Seiryo-machi, Aoba-ku, Sendai, Miyagi, 980-8575, Japan; Department of Radiation Oncology, Tohoku University Graduate School of Medicine, 1-1 Seiryo-machi, Aoba-ku, Sendai, Miyagi, 980-8574, Japan; Department of Radiological Technology, Faculty of Medicine, School of Health Sciences, Tohoku University, 21 Seiryo-machi, Aoba-ku, Sendai, Miyagi, 980-8575, Japan; Department of Radiation Oncology, Tohoku University Graduate School of Medicine, 1-1 Seiryo-machi, Aoba-ku, Sendai, Miyagi, 980-8574, Japan; Department of Radiation Technology, Tohoku University Hospital, 1-1 Seiryo-machi, Aoba-ku, Sendai, Miyagi, 980-8574, Japan; Department of Radiation Oncology, Tohoku University Graduate School of Medicine, 1-1 Seiryo-machi, Aoba-ku, Sendai, Miyagi, 980-8574, Japan; Department of Radiation Oncology, Tohoku University Graduate School of Medicine, 1-1 Seiryo-machi, Aoba-ku, Sendai, Miyagi, 980-8574, Japan; Department of Radiation Oncology, Tohoku University Graduate School of Medicine, 1-1 Seiryo-machi, Aoba-ku, Sendai, Miyagi, 980-8574, Japan; Department of Radiological Technology, Faculty of Medicine, School of Health Sciences, Tohoku University, 21 Seiryo-machi, Aoba-ku, Sendai, Miyagi, 980-8575, Japan

**Keywords:** radiotherapy, head and neck cancer, machine-learning algorithms, volume reduction

## Abstract

In external radiotherapy of head and neck (HN) cancers, the reduction of irradiation accuracy due to HN volume reduction often causes a problem. Adaptive radiotherapy (ART) can effectively solve this problem; however, its application to all cases is impractical because of cost and time. Therefore, finding priority cases is essential. This study aimed to predict patients with HN cancers are more likely to need ART based on a quantitative measure of large HN volume reduction and evaluate model accuracy. The study included 172 cases of patients with HN cancer who received external irradiation. The HN volume was calculated using cone-beam computed tomography (CT) for irradiation-guided radiotherapy for all treatment fractions and classified into two groups: cases with a large reduction in the HN volume and cases without a large reduction. Radiomic features were extracted from the primary gross tumor volume (GTV) and nodal GTV of the planning CT. To develop the prediction model, four feature selection methods and two machine-learning algorithms were tested. Predictive performance was evaluated by the area under the curve (AUC), accuracy, sensitivity and specificity. Predictive performance was the highest for the random forest, with an AUC of 0.662. Furthermore, its accuracy, sensitivity and specificity were 0.692, 0.700 and 0.813, respectively. Selected features included radiomic features of the primary GTV, human papillomavirus in oropharyngeal cancer and the implementation of chemotherapy; thus, these features might be related to HN volume change. Our model suggested the potential to predict ART requirements based on HN volume reduction .

## INTRODUCTION

Radiation therapy is as effective as surgery and chemotherapy in the treatment of head and neck (HN) cancer because nearly all HN cancers are squamous cell carcinomas (SCC), which are relatively radiosensitive, and conservative treatment is a major factor in improving the quality of life (QOL) [1]. Standard radiotherapy techniques for the treatment of HN SCC are highly conformal, modulated techniques, such as intensity-modulated radiation therapy (IMRT), helical IMRT (tomotherapy) and volumetric modulated arc therapy (VMAT). These techniques enable the delivery of high radiation doses to tumor volumes while minimizing the dose to surrounding structures and reducing normal tissue toxicities such as xerostomia [2, 3]. However, during radiotherapy, many patients will experience volumetric and spatial changes in the target volumes and organs at risk (OAR), which may be due to the combination of treatment response, inflammation, muscle atrophy and radiation effects on normal tissues [1]. Patients with HN cancer tend to lose weight during radiation therapy, typically 5–15% from their initial bodyweight [4, 5]. The clinical target volume (CTV) is also reduced, with up to a 10% reduction within 2 weeks of treatment [4]. The parotid gland volume was reported to decrease by 15% within 2 weeks and up to 35% by the end of irradiation, and it has also been reported to undergo positional changes of 1–3 mm inward or outward [6]. These anatomical or geometric changes increase the delivered dose to normal tissues or decrease the dose to gross tumor volume (GTV) during the treatment course [7, 8]. In addition, the prediction of HN volume changes may be useful for heavy particle therapy because changes in the beam depth have greater effects on dose distribution than photon beam radiotherapy.

Adaptive radiation therapy (ART) has the potential to resolve issues such as GTV and OAR dose changes caused by anatomic changes [9–11], and studies have reported that ART can improve tumor control and QOL [9, 10, 12]. Various ART protocols are currently under investigation and have been reported to be effective for HN cancer [13, 14]. However, ART requires periodic rescanning, recontouring, replanning and plan validation, which requires high costs and effort [8, 15, 16]. Many uncertainties about ART remain, such as the need for ART, consideration of patient background in whom ART should be performed and the most appropriate number of replans and their timing. Yu *et al*. [12] and Alves *et al*. [17] have suggested that certain radiomic features may predict patients with HN cancer who require ART. However, these studies are likely not quantitative because the selection of cases for ART depends on the decision of the physician, and other institutions and other physicians may make different decisions. As a quantitative measure, we focused on changes in the HN volume.

Cone-beam computed tomography (CBCT) is used for image-guided radiotherapy (IGRT) in all treatment fractions in our hospital, and the HN volume can be calculated from them. In some previous studies, the contour of specific areas, as well as the circumference and diameter of the HN were used as indices to investigate anatomical changes [18, 19]. However, these measurements were only one part of the HN and may have missing anatomical changes in the contour of a specific site, and we thought that the HN volume would be more likely to experience anatomical changes. To the best of our knowledge, no studies have reported HN volume calculations from CBCTs for all fractions of radiotherapy. A study reported that a 10-mm loss in the neck average radius corresponded to an increment in the dose to the OARs of up to 4% [20]. Thus, an association between HN volume reduction and dose changes may exist.

This study aimed to predict patients with HN cancer who will more likely require ART based on a quantitative measure of HN volume reduction using clinical factors, dose–volume histogram (DVH) parameters and radiomic features. This study may be the first to predict HN volume changes that occur during external irradiation.

## MATERIALS AND METHODS

### Patient characteristics

In total, 172 of 255 patients who received radiotherapy to the HN region at our Hospital between August 2018 and June 2021 were retrospectively enrolled in this study. The exclusion criteria were as follows: primary tumor was not HN cancer (*n* = 6), treatment with three-dimensional (3D) conformal radiotherapy (*n* = 10), patients who underwent surgery with neither primary gross tumor volume (GTVp) nor nodal GTV (GTVn) (*n* = 23), inadequate contouring (*n* = 2), treatment with intra-arterial injection chemotherapy (*n* = 4), sever CT artifact and lack of CBCT range (*n* = 38). All cases received radiation therapy for radical or postoperative irradiation. These cases underwent CBCT for IGRT at all treatment fractions. The tumor sites were the nasopharynx, mesopharynx, hypopharynx, oral cavity, larynx and paranasal sinuses. Other patient details are shown in Table 1. This study was approved by Tohoku University institutional research ethics committee (Approval No. 2021-1-1011). The data used in this study has not been made publicly available.

**Table 1:** Patient information

Patient information	Category	Total (*n* = 172)
Gender	Male	144 (84%)
	Female	28 (16%)
Pathology	SCC	164 (95%)
	Except SCC	8 (5%)
Primary site	Pharynx	108 (63%)
	Oral cavity	33 (19%)
	Larynx	23 (13%)
	Other	8 (5%)
Oropharynx carcinoma HPV16	Positive	26 (59%)
	Negative	18 (41%)
Treatment	Radical radiotherapy	128 (74%)
	Postoperative recurrence radiotherapy	44 (26%)
Chemotherapy	Done (CDDP)	111 (65%)
	Done (except CDDP)	8 (5%)
	No	53 (31%)
GTV	Only GTVp	37 (22%)
	Only GTVn	32 (19%)
	Both	103 (60%)
Patient information	Median (range)	
Age	67 (28–90)	
Body weight(kg)	59.9 (31–93.3)	
Body mass index (kg/m^2^)	22.2 (14.3–31.9)	
Total protein (g/dl)	6.8 (5.1–8.3)	
Albumin (g/dl)	3.8 (2.0–4.7)	
Albumin/globulin ratio	1.3 (0.4–2.1)	
Hemoglobin (g/dl)	13.3 (8.7–17.7)	

### Treatment planning

All patients underwent planning CT (pCT) with 2–2.5 mm slice sickness and 1.17–1.27 mm pixel size using SOMATOM Definition AS+ system (SIEMENSE, Munich, Germany). At our institution, a resident doctor and an experienced radiation oncologist collaboratively performed the ROI contouring per patient. Moreover, each radiotherapy plan and ROI contouring were evaluated during a conference involving the Department of Radiation Oncology and Head and Neck Surgery. With the validation of both departments during the conference, we aim to minimize bias by radiation oncologist in contouring as much as possible. All plans were created with Monaco version 5.0 (Elekta, Stockholm, Sweden), and the Monte Carlo method was used as the dose calculation algorithm. All patients were treated by Versa HD (Elekta, Stockholm, Sweden). Our hospital adopts a two-step method, in which the patient is scanned again during radiotherapy, and a boost plan is created based on the rescanned pCT. For initial treatment, 44 Gy/22 fractions (or 40 Gy/20 fractions) were prescribed for the CTV primary, CTV nodal and CTV prophylactic areas plus a 5-mm PTV margin. For boost treatment, 26 Gy/13 fractions (or 22 Gy/11 fractions for postoperative patients) were prescribed in the CTV primary and CTV nodal regions plus a 5-mm PTV margin.

### Calculation and classification method of the HN volume

**Fig. 1:**
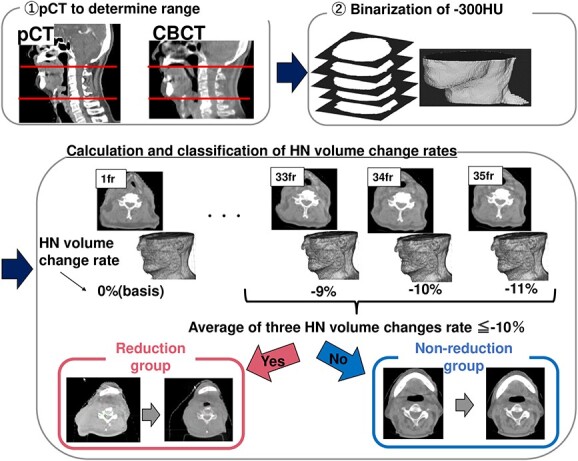
Calculation and classification method of the HN volume. First, the slices were defined by visually checking the range of C1–C5 spinous process on the pCT. Then, the levels defined in the CT were searched on the CBCT, and the range was binarized using in-house software to calculate the volume. The patients whose HN volume was <−10% in the average of the minimum fraction and the three fractions before and after the minimum fraction during the irradiation course were defined as the reduction group, and the others were defined as the non-reduction group.

The method of calculating and classifying the HN volume is shown in Fig. 1. The HN volume was calculated using CBCT for IGRT for all treatment fractions and calculated using an in-house program created in MATLAB 2021b (MathWorks, Natick, MA, USA). In our hospital, CBCT images were acquired for all treatment fractions and then matched with the pCT, and the differences in coordinates between them were saved into the registration file. These data were used to calculate the HN volume. The imaging conditions for all fractions of all patients were identical to the imaging conditions for all fractions of all patients as follows: 100 kV, 36.6 mAs and 270 mm FOV. These were acquired using an X-ray positioning system, X-ray Volume Imaging (Elekta, Stockholm, Sweden), with a pixel size of 1 mm × 1 mm and a slice thickness of 3 mm.

First, the first cervical spinous process level (C1) and the fifth cervical spinous process level (C5) level were determined by the radiological technologist with advice from an experienced radiation oncologist to establish the range on the pCT. Then, two datasets were acquired: the slices that specified the C1 and C5 levels in the pCT, and the registration file that stored the movement of the bed for positioning. The two CBCT slices closest to the slice coordinates specified in the pCT for treatment planning were identified. To correspond to the shift interval of the registration file, interpolation was made between two CBCT slices in 0.01-mm increments. It may reduce errors related to the range because registration files were used to determine the same position. After specifying the range, a cropping range was set on CBCT to exclude the treatment couch and immobilization devices in the HN region. Furthermore, the pCT images were converted to binary images based on the threshold values, and holes created during the binarization were filled by morphological expansion and contraction operations. This was done to ensure that the holes did not underestimate the HN volume. Finally, the HN volume was obtained by multiplying the voxel volume determined from the pixel spacing (1 mm) and slice thickness (3 mm) of the CBCT by the number of voxels within the HN body contour, and the volume change rate $\left(\Delta V\right)$ was defined as follows:


$$ \Delta V=\left|\frac{V_n-{V}_{1 st}}{V_{1 st}\ }\right|\left(\%\right) $$

where ${V}_n$ is the HN volume in the n fraction and ${V}_{1 st}$ is the corresponding value in the first fraction.

When the $\Delta V$ became >5% between successive fractions, we checked the binarized image. If the binarized image had a problem, such as the inclusion of the immobilization device, adjustments were made to the threshold of binarization and pixel removal. When this adjustment occurred, the $\Delta V$ was recalculated for all other fractions.

In this study, the threshold for the reduction and non-reduction groups was defined as a reduction rate of at least 10% in three consecutive fractions. Studies have reported that 21–66% of patients benefit from ART [13, 21]. Therefore, the threshold in this study was defined as the top 30% with a large reduction in the HN volume change rate, which corresponds to −10% of the HN volume change. Three consecutive fractions were used because a single fraction was likely to include uncertainty in the calculation of the HN volume, and the average of three fractions reduced this effect. In this way, all cases were separated into the reduction group and the non-reduction group. An example of how to count fractions is shown below. If a patient shrank after 24, 25 or 26 fractions, the number was the 25th fraction.

### Feature extraction

#### Radiomic feature extraction

**Fig. 2:**
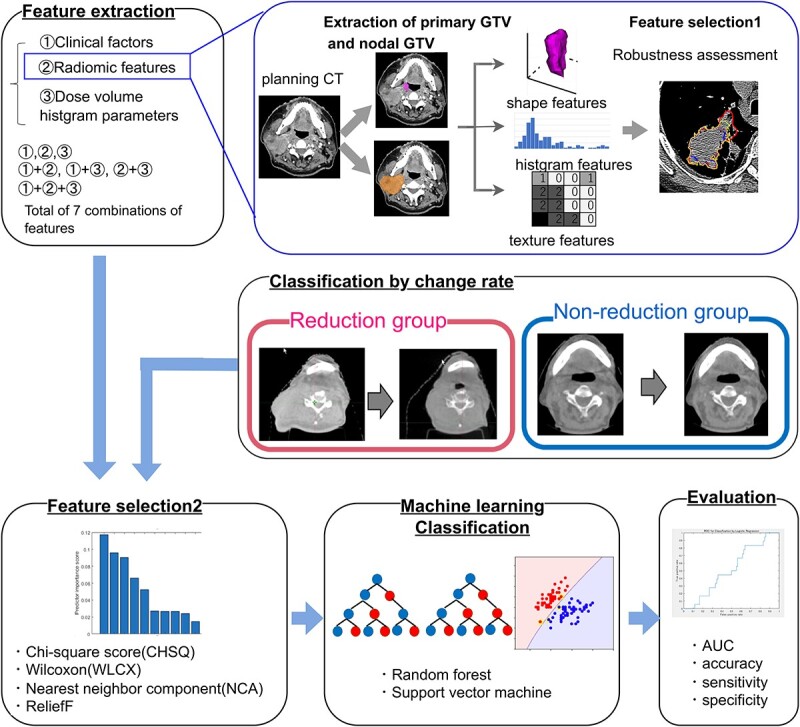
Workflow of this study. *GTV, gross tumor volume. Using clinical factors, dose parameters and radiomic features, eight models for predicting HN volume reduction were constructed using four feature selections and two machine-learning algorithms, and the prediction accuracy for each model was evaluated.

Figure 2 shows the overall workflow of this study and the extraction flow of the radiomic features. The extraction of radiomic features was implemented in PyRadiomics software Version 2.2.0 [22] with 3D Slicer Version 4.10.2. After resampling to a pixel size of 1 × 1 × 1 mm^3^ in order to reduce feature variation, the features were extracted from each GTVp and GTVn without any filtering (14 shape features, 18 first-order features, and 75 texture features). Texture features were extracted belonging to the following categories: gray-level co-occurrence matrix (GLCM), gray-level run-length matrix, gray-level size zone matrix, neighborhood gray-tone difference matrix, gray-level gap length matrix, neighboring gray-level dependence matrix and gray-level distance zone matrix. PyRadiomics was used because most radiomic features are based on imaging biomarker standardization initiative.

#### Clinical factors and DVH parameters

All clinical factors are listed in Table 1. The PTV volume used the values from Monaco, a treatment planning system. The dose parameters were calculated from the dose distribution obtained by dose summation of the initial plan (40 Gy/44 Gy) and boost plan (30 Gy/26 Gy). The dose distribution was created by accumulating the dose recalculated on the boost CT without changing the initial plan segment and monitor unit , and the boost plan dose was then converted to dose per irradiation. A total of nine dose parameters were calculated: D_2%_, D_50%_ and D_98%_ for each of GTVp, GTVn and PTV.

#### Feature selection

Radiomic features vary with contouring [23], and multiple segmentation was applied to select robust features. This method evaluates the interobserver variability of the segmentations. Based on previous studies [23–25], we extracted radiomic features using publicly available data from The Cancer Imaging Archive [26] and evaluated their robustness using the intraclass correlation coefficient (ICC) with MATLAB. Robustness was evaluated using the ICC for Case 3A [26]. Features with ICC > 0.7 were considered robust, and those that were not robust were not used in the feature selection stage of the algorithm. Features with values that varied significantly with slight differences in segmentation were excluded because they were not considered sufficiently robust. Table 2 shows all the radiomic features and features with ICC > 0.7.

**Table 2:** All radiomic features used in this study (*n* = 107)

Category	Feature name	ICC > 0.7
Shape	Voxel volume	*
Shape	Maximum 3D diameter	*
Shape	Mesh volume	*
Shape	Major axis length	*
Shape	Sphericity	0
Shape	Least axis length	*
Shape	Elongation	0
Shape	Surface volume ratio	*
Shape	Maximum 2D diameter slice	*
Shape	Flatness	0
Shape	Surface area	*
Shape	Minor axis length	*
Shape	Maximum 2D diameter column	*
Shape	Maximum 2D diameter row	*
Intensity	Interquartile range	0
Intensity	Skewness	*
Intensity	Uniformity	0
Intensity	Median	*
Intensity	Energy	*
Intensity	Robust mean absolute deviation	*
Intensity	Mean absolute deviation	*
Intensity	Total energy	*
Intensity	Maximum	*
Intensity	Root mean squared	*
Intensity	90th percentile	*
Intensity	Minimum	*
Intensity	Entropy	*
Intensity	Range	0
Intensity	Variance	*
Intensity	10th percentile	*
Intensity	Kurtosis	*
Intensity	Mean	0
Texture (GLCM)	Joint average	*
Texture (GLCM)	Sum average	*
Texture (GLCM)	Joint entropy	*
Texture (GLCM)	Cluster shade	*
Texture (GLCM)	Maximum probability	*
Texture (GLCM)	Inverse difference moment normalized	*
Texture (GLCM)	Joint energy	*
Texture (GLCM)	Contrast	*
Texture (GLCM)	Difference entropy	*
Texture (GLCM)	Inverse variance	*
Texture (GLCM)	Difference variance	*
Texture (GLCM)	Inverse difference normalized	0
Texture (GLCM)	Inverse difference moment	*
Texture (GLCM)	Correlation	0
Texture (GLCM)	Autocorrelation	0
Texture (GLCM)	Sum entropy	*
Texture (GLCM)	Maximal correlation coefficient	*
Texture (GLCM)	Sum squares	*
Texture (GLCM)	Cluster prominence	*
Texture (GLCM)	Informational measure of correlation 1	0
Texture (GLCM)	Informational measure of correlation 2	0
Texture (GLCM)	Difference average	*
Texture (GLCM)	Inverse difference	*
Texture (GLCM)	Cluster tendency	*
Texture (GLRLM)	Short run low gray-level emphasis	0
Texture (GLRLM)	Gray-level variance	0
Texture (GLRLM)	Low gray-level run emphasis	0
Texture (GLRLM)	Gray-level nonuniformity normalized	0
Texture (GLRLM)	Run variance	0
Texture (GLRLM)	Gray-level nonuniformity	0
Texture (GLRLM)	Long run emphasis	*
Texture (GLRLM)	Short run high gray-level emphasis	0
Texture (GLRLM)	Run length nonuniformity	*
Texture (GLRLM)	Short run emphasis	*
Texture (GLRLM)	Long run high gray-level emphasis	0
Texture (GLRLM)	Run percentage	0
Texture (GLRLM)	Long run low gray-level emphasis	*
Texture (GLRLM)	Run entropy	*
Texture (GLRLM)	High gray-level run emphasis	0
Texture (GLRLM)	Run length nonuniformity normalized	0
Texture (GLSZM)	Gray-level variance	0
Texture (GLSZM)	Zone variance	*
Texture (GLSZM)	Gray-level nonuniformity normalized	*
Texture (GLSZM)	Size zone nonuniformity normalized	*
Texture (GLSZM)	Size zone nonuniformity	*
Texture (GLSZM)	Gray-level nonuniformity	*
Texture (GLSZM)	Large area emphasis	*
Texture (GLSZM)	Small area high gray-level emphasis	*
Texture (GLSZM)	Zone percentage	*
Texture (GLSZM)	Large area low gray-level emphasis	*
Texture (GLSZM)	Large area high gray-level emphasis	0
Texture (GLSZM)	High gray-level zone emphasis	0
Texture (GLSZM)	Small area emphasis	*
Texture (GLSZM)	Low gray-level zone emphasis	*
Texture (GLSZM)	Zone entropy	0
Texture (GLSZM)	Small area low gray-level emphasis	*
Texture (GLDM)	Gray-level variance	0
Texture (GLDM)	High gray-level emphasis	*
Texture (GLDM)	Dependence entropy	*
Texture (GLDM)	Dependence nonuniformity	*
Texture (GLDM)	Gray-level nonuniformity	*
Texture (GLDM)	Small dependence emphasis	*
Texture (GLDM)	Small dependence high gray-level emphasis	*
Texture (GLDM)	Dependence nonuniformity normalized	*
Texture (GLDM)	Large dependence emphasis	*
Texture (GLDM)	Large dependence low gray-level emphasis	*
Texture (GLDM)	Dependence variance	*
Texture (GLDM)	Large dependence high gray-level emphasis	0
Texture (GLDM)	Small dependence low gray-level emphasis	0
Texture (GLDM)	Low gray-level emphasis	0
Texture (NGTDM)	Coarseness	*
Texture (NGTDM)	Complexity	*
Texture (NGTDM)	Strength	*
Texture (NGTDM)	Contrast	0
Texture (NGTDM)	Busyness	*

GLCM = gray-level co-occurrence matrix, GLDM = gray-level dependence matrix, GLRLM = gray-level run length matrix, GLSZM = gray-level size zone matrix, NGTDM = neighboring gray tone difference matrix. In the rightmost column, features with ICC > 0.7 (robust features) are marked with “*”, other features are marked with “-”.

Then, an algorithm-based feature selection was used to remove redundant or irrelevant features. To optimize model performance, investigating different feature selection methods is necessary because the final prediction accuracy is expected to vary depending on the choice of algorithm. The used methods were specifically chosen for their applicability to the binary classification problem. In this study, four filter-type feature selection methods were used to rank the features: Chi-square score (CHSQ), minimum redundancy maximum relevance (MRMR), neighborhood component analysis and relief. Finally, each selection method was used to select the top 10 features according to the rank. A study recommended reducing the number of features to one-tenth of the event in the test data, and with this as a reference, the number of features was 5 in this study, and the values around that (1–10) were examined [27].

#### Building predictive models

**Fig. 3:**
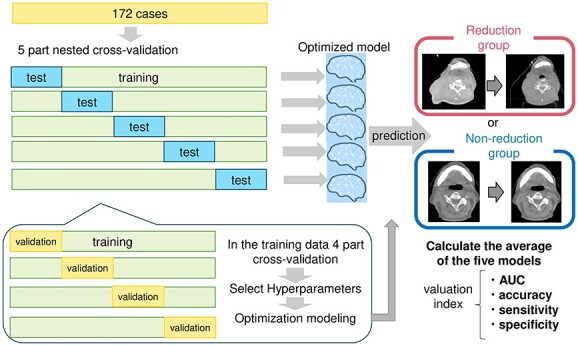
Prediction model for HN volume change rate using machine learning. A five-part nested cross-validation was performed. In this cross-validation, a four-part cross-validation was performed, and the hyperparameters with the highest accuracy were used in the training data. The prediction accuracy was calculated by inserting the test data into the model optimized with the training data. Since the accuracy of the five models was calculated by cross-validation, the average of the accuracy of the five models was used as the evaluation index.

Figure 3 shows an overview of machine learning. The input data of the predictive model were a feature set of seven different combinations of clinical factors, radiomic features and dose parameters, and these seven combinations are also shown in Fig. 2. For each of the seven feature sets, the top 10 features were selected using each feature selection method. Accuracy evaluation was performed by the nested 5-fold cross-validation. First, all cases were randomly divided into five parts, one set of which was used as the test data. At that time, data were divided so that the proportions of the reduction and non-reduction groups were of the same rate in each data set. Then, 5-fold cross-validation was performed among the remaining four sets to optimize the hyperparameters for machine learning. The test data were then put into the optimized model, and the accuracy was calculated as described later. The accuracy of the average of the five models was used as the final result. The area under the curve (AUC) was investigated by increasing the number of features in each feature set from 1 to 10, starting with the most importance feature for each of the nested 5-fold cross-validation. In that feature set, the model with the highest AUC was taken as the representative model.

Two machine-learning algorithms, random forest (RF) and support vector machine (SVM), were considered. Schikit-learn version 1.1.1 was used for implementation. The parameters for RF and SVM were determined to have the highest ‘balanced accuracy’ on the training data. The following hyperparameters of RF were explored: (i) criterion: gini/entropy; (ii) decision tree depth: 3–11; and (iii) number of decision trees: 10–2000. The following hyperparameters of SVM were explored: (a) cost parameter to determine how much misclassification to tolerate: 1–1000; (b) kernel type: liner/rbf/poly/sigmoid; (c) gamma: 0.001–0.0001. Balanced accuracy can evaluate the performance on unbalanced data more appropriately than ‘Accuracy’. ‘Balanced accuracy’ was defined as follows:


$$ blanced\ accuracy=\frac{1}{2}\left(\frac{TP}{TP+ FN}+\frac{TN}{TN+ FP}\right) $$

where $TP$ is true positive, $FN$ is false negative, $TN$ is true negative and $FP$ is false positive. The model was built after the parameters were determined in this manner. Finally, the AUC, accuracy, sensitivity and specificity of the predictive model in the test data for the HN volume change rate were evaluated.

## RESULTS

### HN volume change rate during the treatment course

In this study, 50 of 172 patients (29%) had a volume reduction of >10%. The mean of the three smallest HN volume changes in each case was calculated to have a median (range) of −4.93% (−19.4 to 0.78%) for all cases, −12.2% (−19.4 to −10.1%) for the reduction groups and − 5.67% (−9.9 to 0.78%) for the non-reduction groups. The median number of fractions with three consecutive HN volume changes of −10% or less was 30 (10–34 fractions).

### Evaluation of HN volume change rate prediction models and feature types

**Fig. 4:**
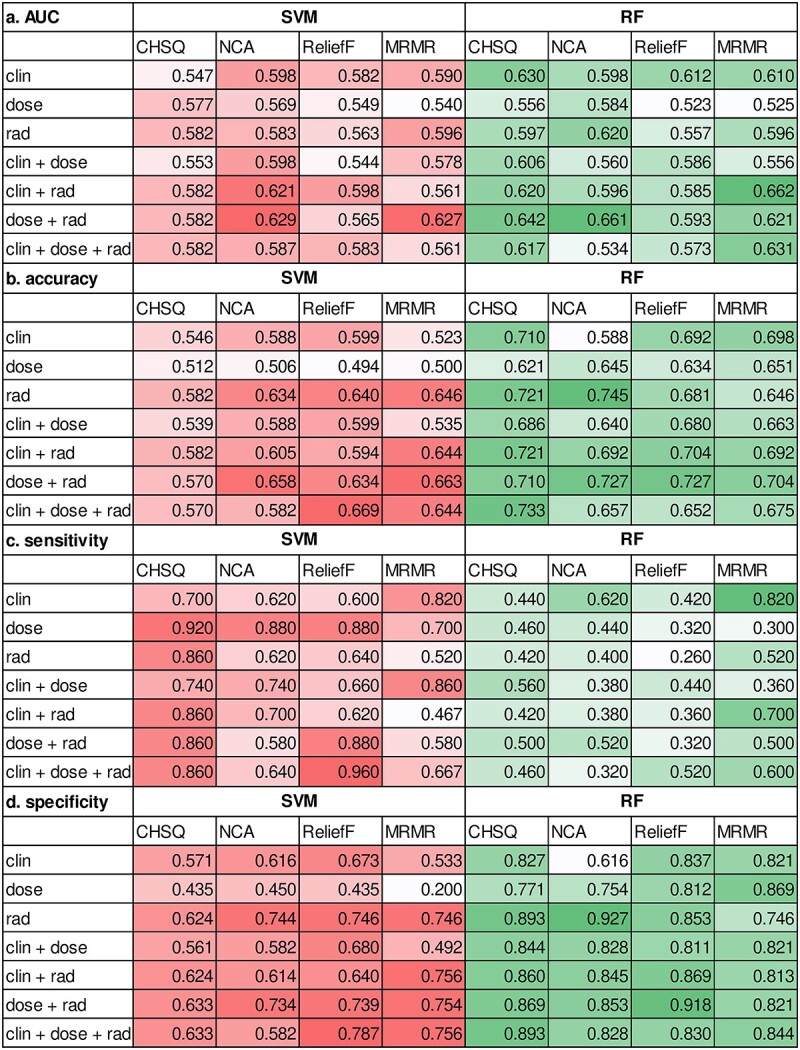
Results for each evaluation index for all cases. (**a**–**d**) Results of the area under the curve, accuracy, sensitivity and specificity. *SVM, support vector machine; RF, random forest; clin, clinical factor; dose, dose parameter; rad, radiomic feature; CHSQ, chi-square score; NCA, neighborhood component analysis; MRMR, minimum redundancy maximum relevance. The AUC, accuracy, sensitivity and specificity are shown for each evaluation index. The left side indicates the SVM, and the right side indicates the RF results; the higher the value, the darker the color. The highest AUC was 0.662, where the feature type was clinical factor + radiomic features, the feature selection was minimum redundancy maximum relevance (MRMR) and the machine-learning algorithm was RF.

All results for each evaluation index are shown in Fig. 4. The highest AUC was 0.662, where the feature set was clinical factor + radiomic features, the feature selection was MRMR and the machine-learning algorithm was RF. The three features used with the highest AUC were informational measure of correlation (IMC)1, which is one of the GLCMs of GTVp and expresses textural complexity, human papillomavirus (HPV) 16 of oropharyngeal cancer and implementation of chemotherapy. The significance levels were 0.7, 0.2 and 0.1 for IMC1, chemotherapy and HPV, respectively. The accuracy, sensitivity and specificity for the model with the highest AUC were 0.692, 0.700 and 0.813, respectively. The RF model parameters that showed the highest AUC were as follows: (1) criterion: entropy; (2) depth of decision tree: 3; and (3) number of decision trees: 10. The AUC changed by up to 0.2 as the test data changed. The difference in AUCs by the test data was the respective AUCs that were cross-validated. The difference in the AUC due to the four different feature selection algorithms was <0.1. Furthermore, the difference in the AUC due to the two machine-learning algorithms was also <0.1. The difference in the AUC by the feature set was a maximum of 0.14. The respective differences between the machine-learning algorithm, feature selection and feature set were obtained by comparing the mean AUCs of the five models.

## DISCUSSION

The highest AUC was 0.662, and the feature set was clinical factor + radiomic features. In that case, the accuracy, sensitivity and specificity were 0.692, 0.700, and 0.813, respectively, and it would not bias the prediction toward either the reduction or non-reduction group. The specific types of features used with the highest AUC were IMC1, which is one of the GLCMs of GTVp and show textural complexity, HPV positivity/negativity for oropharyngeal cancer and implementation of chemotherapy (*cis*-diamminedichloro-platinum, CDDP). The significance levels were 0.7 for IMC1, 0.2 for chemotherapy and 0.1 for HPV. A study reported that tumor complexity is associated with treatment-resistance [28]. We investigated the IMC1 values for the reduced and non-reduced groups. The results showed less heterogeneity in the reduced group (IMC1 mean = −0.35) and greater heterogeneity in the non-reduced group (IMC1 mean = −0.47) (the closer to −1, the greater the heterogeneity). Therefore, we hypothesized that the large complexity might indicate the presence of treatment-resistant areas within the tumor, which might result in nonreduction tumors. In addition, chemotherapy with CDDP is known to cause radiosensitizing affects. Thus, chemotherapy-treated patients were more affected by radiation and lost weight because of taste disturbance, nausea and decreased gastrointestinal tract function and had greater HN volume reduction [28]. Silander *et al*. [28] reported that >66% of patients with HN cancer who received treatment lost ≥10% of their bodyweight, primarily due to fat loss, and this was pronounced in patients who received chemotherapy. Patients with HPV-positive oropharyngeal cancer have high radiosensitivity and extensive lymph node involvement [29], and the extensive PTV reduction may have contributed to the HN volume reduction. Regarding the predictors of ART, to our knowledge, no study has predicted HN volume reduction; however, some previous studies have predicted tumor volume reduction. Surucu *et al*. [8] concluded that chemotherapy, age and tumor site are important predictors of GTVp shrinkage, whereas performance status, primary site and age are important predictors of GTVn shrinkage. In this study, the clinical factors that resulted in the largest AUCs in the HN volume reduction model were HPV and chemotherapy for oropharyngeal cancer, consistent only with the chemotherapy proposed by their study. Performance status was not examined in this study, and the primary site may not have been chosen as a useful factor because of many mixed primary sites. On the contrary, no dose parameters were selected because dose constraints were applied to D_min_, D_95%_ and D_2%_ of the PTV during optimization, and case differences were small for the dose parameters used in this study.

In feature selection, the model made with features selected by MRMR showed the highest AUC. A study reported that MRMR has better stability for feature selection methods [30]; therefore, it might have been useful in this study [31]. However, the difference in the AUC by feature selection was <0.1. The machine-learning algorithm had the highest AUC for RF, and the difference in the AUC due to the two types of machine-learning algorithms was also <0.1 in this study. Therefore, the difference due to the type of machine learning and features might be small. Within 5-fold cross-validation, the AUC changed by a maximum of ~0.2 when the test data were replaced. The reduction and non-reduction groups were divided in the same proportions, and no significant bias was found in all the features selected in the model with higher AUC. Therefore, this was not due to feature bias in each dataset, and the failure to optimize the model may be caused by other factors. Some features might not have been captured, which were the cause of the HN volume reduction, and these features were biased. In other words, some cases may be good at prediction and some may not, and these may have been biased in each dataset.

Although no previous studies have used the HN volume as an indication criterion for ART, some previous studies have predicted actual ART cases based on clinical factors and radiomic features. Alves *et al*. [17] used SVM and reported accuracy and AUC of 0.821 and 0.843, respectively, which were higher than those reported in the present study. Yu *et al*. [12] also reported that the radiomic features could predict ART and non-ART groups of patients with nasopharyngeal cancer with high accuracy (AUC = 0.93). This study was less accurate than previous studies because of several reasons.

First, the ART application criteria were different. Both studies have used criteria such as weight loss, lymph node shrinkage, cervical tissue loss and cervical contour discrepancy, whereas in the present study, the sole criterion for prediction was HN volume reduction. Previous studies have suggested thresholds for selecting patients for ART, namely, head contour change >1 cm, weight loss >10% and overdose to tumor >5% [31], and the HN volume may be only associated with head contour and weight loss. Yao *et al*. [32] reported that the weight-loss rates had a moderate to strong correlation with skin separation (decrease from the original contour of the body surface) reductions at C1, C2 and C4 levels in their article on replanning criteria. In this study, weight data were obtained only for inpatients; thus, analyzing the weight of all patients was difficult. In addition, we could not determine the position of the HN contour (slice) to be investigated because the study included HN cancers of various primary sites; thus, we did not analyze the head contour change. The prediction of ART suitability could be improved by including not only HN volume reduction but also primary and lymph node metastasis reduction, and other factors.

Second, the factors utilized in this study may not be informative enough to predict the HN volume. Radiomic features of GTVp and GTVn were used to predict tumor shrinkage, and these were reported to be useful [28]. However, in some cases, HN volume reduction might be due to not only tumor shrinkage but also fats, muscles and some OARs, like the parotid grand, and in these cases, predicting shrinkage using radiomic features of the GTV appears difficult. Non-tumor radiomic features such as fats and muscles in the HN region would be useful for prediction but were not possible because of contouring difficulties. In addition, radiomic features of OARs were not investigated in this study because various disease sites were included in the studied cases, none of which were commonly included in the irradiated area. The same can be considered for dose parameters. Although target-related dose parameters were used in this study, parameters for OARs such as the parotid gland and spinal cord may be more effective. However, they were not investigated in this study for the same reasons as for radiomic features.

This study has several limitations. First, the calculation method was uncertain, and the HN volume had a narrow range. It may contain various errors, including narrow ranges, patient rotation errors, effects of the low CBCT image quality and range deviations between initial and boosted CT. Only the range of C1–C5, which is narrower than the irradiation range, was calculated. In addition, the same range was identified in the registration file; however, it may have included the error portion of rotation that cannot be corrected in the patient’s three axes and the effect of noise and artifacts from the CBCT. Moreover, the treatment pCT had an initial and a boost CT, and no information was available between these two, so the same slices were identified visually.

Second, the relationship between changes in the HN volume and dose changes is unclear. A study reported that a decrease in the HN volume increases the OAR dose and decreases the target dose coverage [20]. We attempted to investigate this using CBCT. However, we gave up the idea because of the uncertain accuracy of dose calculation due to the differences in image quality and CT values, the lack of contours for each ROI on the CBCT and the low accuracy even if deformable image registration was used. Therefore, the threshold setting was not determined from the perspective of dose changes but from the percentage of cases with ART indications in the HN area, and the threshold setting was set at −10% [20]. Even with comparable anatomical changes, dose changes are likely to depend on the treatment plan [33] (e.g. a plan with one round of VMAT vs a plan with half a round, where one round is more robust to anatomical changes.) The requirement of ART may differ depending on the facility’s treatment plan. For future clinical use, it is essential to clarify the relationship between HN volume change and the resulting dose change of target and OAR.

Third, the feature values were uncertain. Specifically, the reproducibility of the selected features and the clinical significance of radiomic features were unclear. In addition, features were selected on the entire dataset to select the features relevant to HN volume reduction because of the insufficient number of patients. Therefore, the results of this study are internal validations, and the performance obtained may have a positive bias [34]. To avoid this, feature selection should be done with training data only for each case. To confirm the reliability of the features and ensure that these results can be generalized, feature selection and model training must be performed using more training data and validated using an independent external validation dataset. Regarding clinical factors and radiomic features, HPV-related oropharyngeal cancer may also be unreliable, as only 15% (26 cases) of the total 172 cases were HPV. Furthermore, the difficulty in the interpretation of the pathophysiological and clinical significance of the radiomic features remains. This is due to the difficulty in ensuring the reproducibility of radiomic features, sharing data, standardizing various procedures and properly validating them, and large multicenter studies are not often conducted [35, 36]. To incorporate the radiomic features into clinical practice, the clinical relevance of these features must be elucidated.

In the future, a possible way to improve accuracy is to use deep learning with convolutional neural networks for feature extraction [37]. The use of transition learning for feature extraction may provide more information than human-defined features. Furthermore, it may improve the prediction accuracy of HN volume reduction even with a small number of cases at a single institution [37]. Moreover, the accuracy is expected to be higher if the number of cases is increased, and the model is constructed separately for each tumor site. Furthermore, although not directly related to increasing accuracy, increasing the number of cases may ensure that the model’s accuracy is particularly robust [38]. Incorporating CTV radiomic features instead of the GTV may be useful. The expected benefit is to involve the tumor extension and microenvironment, such as the vasculature [39].

In this study, we calculated the HN volume using CBCT images, which were taken during each treatment fraction for IGRT. In addition, the prediction of HN volume change may be useful for proton and heavy particle therapy because changes in the beam range can greatly affect the dose distribution than X-ray therapy. Owing to the need for replanning because of changes in planning doses and the expected increase in the number of treatment plans, this study may be useful in attempting to predict the HN volume.

## CONCLUSION

The combination of clinical factors and GTV radiomic features showed the potential to predict cases of HN volume reduction during VMAT for HN cancer. Further studies are needed to apply the results to the selection of priority cases for ART, starting with an investigation of the association between HN volume reduction and dose distribution changes.
