# Correction to: Development of a prediction model for head and neck volume reduction by clinical factors, dose–volume histogram parameters and radiomics in head and neck cancer

**DOI:** 10.1093/jrr/rrad062

**Published:** 2023-09-06

This is a correction to: Miyu Ishizawa and others, Development of a prediction model for head and neck volume reduction by clinical factors, dose–volume histogram parameters and radiomics in head and neck cancer, *Journal of Radiation Research*, 2023;, rrad052, https://doi.org/10.1093/jrr/rrad052

In the originally published version of this manuscript, there was an error in the affiliation of the first author. This should read: ``Department of Radiological Technology, Faculty of Medicine, School of Health Sciences, Tohoku University, 21 Seiryo-machi, Aoba-ku, Sendai, Miyagi, 980-8575, Japan.'' instead of ``Department of Heavy Particle Medical Science, Graduate School of Medical Science, Yamagata University, 2-2-2 Iidanishi, Yamagata, Yamagata, 990-2331, Japan''. The enumeration and affiliations of all co-authors have been correspondingly adjusted.

These emendations have been made in the article.

